# Supplementary material for: Impacts of Antiretroviral Therapy on the Oral Microbiome and Periodontal Health of Feline Immunodeficiency Virus-Positive Cats
Source: Viruses. 2025 Feb 13;17(2):257. doi: 10.3390/v17020257 (PMC11861066; doi:10.3390/v17020257)
Supplement: Supplementary file 1 [file viruses-17-00257-s001.zip › viruses-3381660-supplementary.pdf]

Supplementary Materials for:

Impacts of Antiretroviral Therapy on the Oral Microbiome and Periodontal Health of Feline Immunodeficiency Virus Positive Cats

Laura Bashor [1], Jennifer E. Rawlinson [2], Christopher P. Kozakiewicz [3,4,5], Elisa Behzadi [1], Craig Miller [6], Jeffrey Kim [7], Megan Cierzan [2], Mary Nehring [1], Scott Carver [8,9,10], Zaid Abdo [1], Sue VandeWoude [1,*]

1. Department of Microbiology, Immunology, and Pathology, Colorado State University, Fort Collins, CO, USA
2. Department of Clinical Sciences, Colorado State University, Fort Collins, CO, USA
3. W.K. Kellogg Biological Station, Michigan State University, Hickory Corners, MI, USA
4. Department of Integrative Biology, Michigan State University, East Lansing, MI, USA
5. Ecology, Evolution, and Behavior Program, Michigan State University, East Lansing, MI, USA
6. Department of Veterinary Pathobiology, College of Veterinary Medicine, Oklahoma State University, Stillwater, OK, USA
7. Comparative Medicine Research Unit, School of Medicine, University of Louisville, Louisville, KY, USA
8. Odum School of Ecology, University of Georgia, Athens, GA
9. Center for the Ecology of Infectious Diseases, University of Georgia, Athens, GA
10. Department of Biological Sciences, University of Tasmania, Tasmania, Australia

* Correspondence: sue.vandewoude@colostate.edu

Figures S1-S3
Table S1

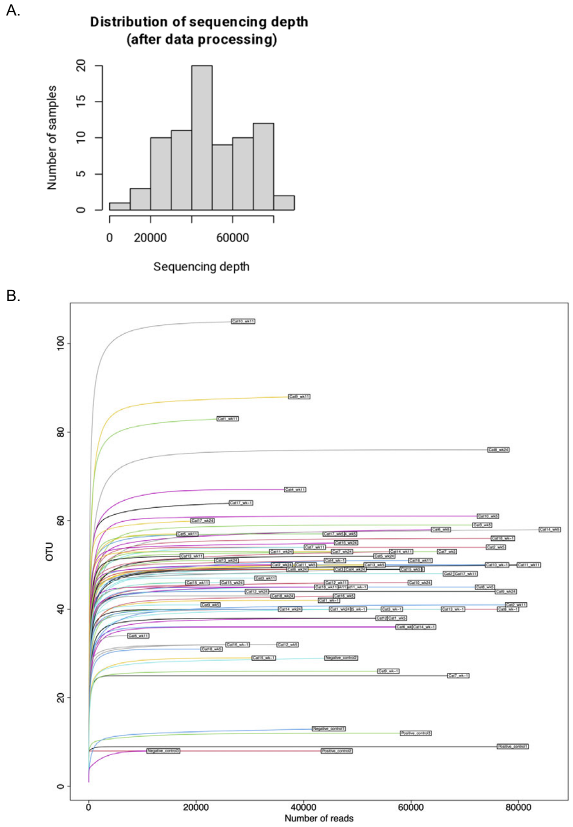

Figure S1. Distribution of sequencing depth (A) by sample and (B) by the number of OTUs identified in a sample. The lowest sequencing depth was obtained from Cat 6 on week 11 (9223); the highest from Cat 14 on week 5.

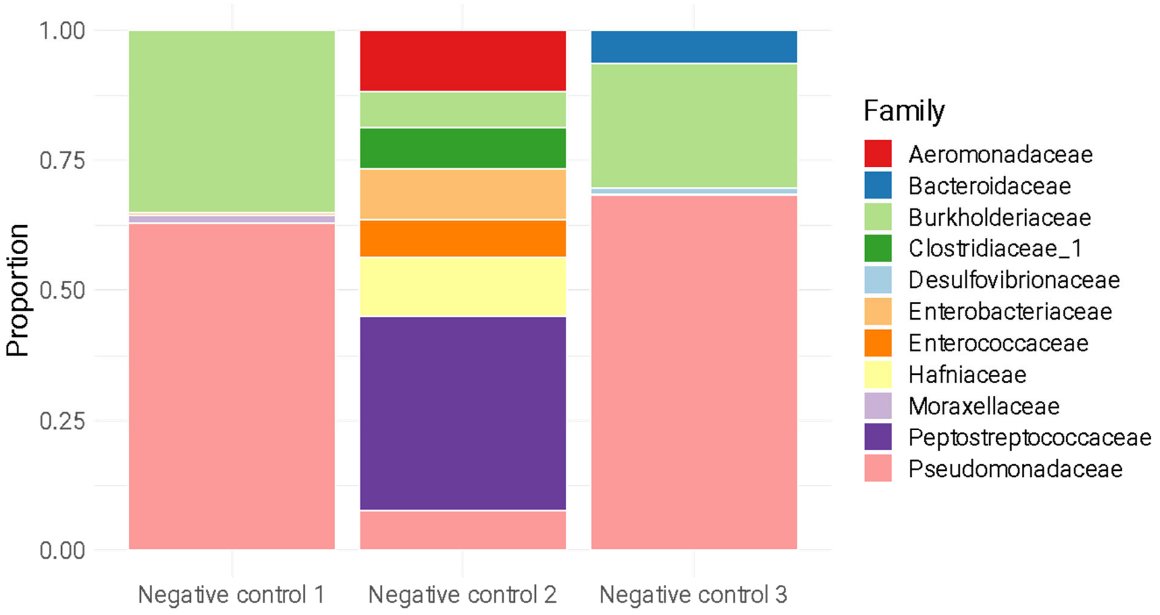

Figure S2. Raw proportions of taxonomic families in the negative control samples for all taxa present in greater than 1% of a sample. Negative control samples were generated via blank DNA extraction and subjected to the same library preparation, sequencing and data processing steps as the other experimental samples.

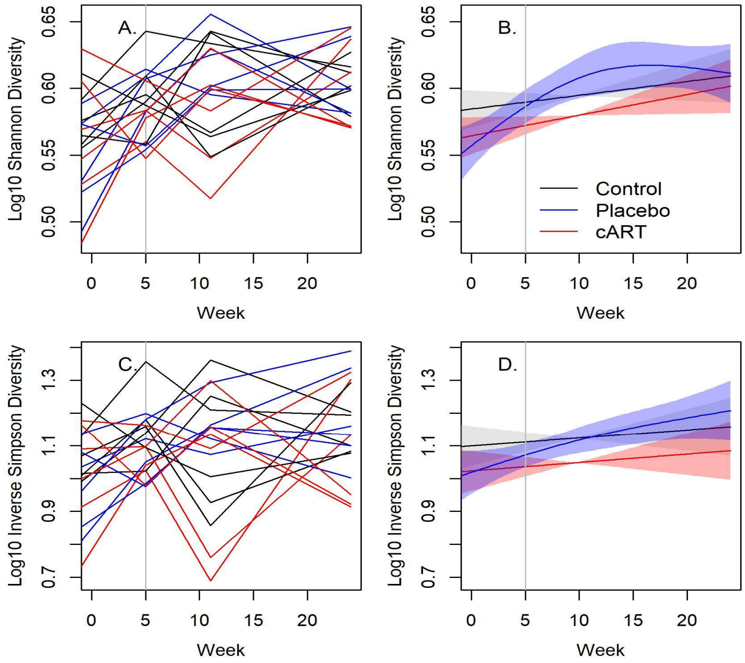

Figure S3. Temporal patterns of two additional measures of alpha diversity (the Shannon Diversity index and the Inverse Simpson Diversity index) of the gingival microbiome of study cats. The Shannon Diversity index and the Inverse Simpson Diversity index were produced by the 'phyloseq' R package. In plots **A** and **C** each line is an individual cat, colored by treatment (black = Control, blue = Placebo, red = cART). In plots **B** and **D**, the associated spline fit from GAM analysis is presented. Vertical line indicates when cART treatment commenced.

Table S1. Alpha diversity measures calculated for each sample included in the study. Diversity measures calculated included the Shannon Diversity index, the Inverse Simpson Diversity index (both produced by the 'phyloseq' package), and the Vegan-Normalized Richness (the expected richness for each sample after normalizing for sequencing depth calculated rarefaction curves produced by the 'vegan' package).

Week	Treatment	Cat	Shannon Diversity Index	Inverse Simpson Diversity Index	Vegan-Normalized Richness
-1	Placebo	Cat7	2.4	8.1	25.0
		Cat8	2.7	11.0	39.2
		Cat9	2.1	5.4	25.7
		Cat10	2.7	9.8	49.2
		Cat11	2.9	12.7	43.6
		Cat12	2.3	6.1	36.8
-1	Control	Cat1	2.7	9.4	41.3
		Cat2	3.1	16.0	47.8
		Cat3	2.8	10.7	39.9
		Cat4	2.9	12.5	49.9
		Cat5	2.6	8.8	39.7
		Cat6	2.6	9.2	35.9
-1	cART	Cat13	2.7	11.3	38.4
		Cat14	2.5	9.0	35.6
		Cat15	2.0	4.4	28.4
		Cat16	2.4	7.2	31.4
		Cat17	3.3	14.0	62.5
		Cat18	3.0	13.6	54.4
5	Placebo	Cat7	3.0	14.2	52.0
		Cat8	2.6	8.4	44.4
		Cat9	2.8	10.2	40.8
		Cat10	3.1	12.2	60.3
		Cat11	3.1	14.8	48.6
		Cat12	2.6	8.6	31.7
5	Control	Cat1	2.6	9.5	37.5
		Cat2	2.9	11.4	53.5
		Cat3	2.9	13.4	57.6
		Cat4	3.4	21.8	56.2
		Cat5	2.9	12.7	47.3
		Cat6	3.1	14.1	55.3
5	cART	Cat13	2.8	11.5	48.3
		Cat14	2.8	11.5	54.6
		Cat15	2.8	10.0	47.9
		Cat16	2.6	9.6	41.7
		Cat17	3.0	13.5	56.5
		Cat18	2.5	8.5	30.9
11	Placebo	Cat7	3.2	18.7	52.7
		Cat8	3.0	13.6	45.0
		Cat9	3.3	13.3	86.1
		Cat10	3.5	10.8	103.3
		Cat11	2.9	12.2	48.1
		Cat12	3.0	13.3	45.1
11	Control	Cat1	3.4	16.9	81.4
		Cat2	2.7	9.1	38.9
		Cat3	2.7	6.2	46.9
		Cat4	3.3	15.2	65.5
		Cat5	3.4	22.0	56.7
		Cat6	2.5	7.5	34.0
11	cART	Cat13	2.5	4.8	51.7
		Cat14	3.3	18.9	50.7
		Cat15	3.0	12.6	45.5
		Cat16	2.3	3.9	48.4
		Cat17	2.8	11.3	47.0
		Cat18	3.0	13.4	43.7
24	Placebo	Cat7	3.4	23.5	52.9
		Cat8	3.4	20.7	73.6

		Cat9	2.8	11.6	43.7
		Cat10	3.0	13.5	44.4
		Cat11	2.8	9.1	52.8
		Cat12	3.0	12.6	43.7
24	Control	Cat1	2.8	11.6	39.2
		Cat2	3.0	10.9	49.1
		Cat3	3.2	18.6	48.7
		Cat4	3.1	14.6	47.3
		Cat5	3.1	15.0	50.5
		Cat6	3.0	11.1	48.6
24	cART	Cat13	3.1	12.6	50.2
		Cat14	2.7	7.9	39.6
		Cat15	2.7	7.2	45.5
		Cat16	3.3	19.1	54.0
		Cat17	3.4	20.1	59.2
		Cat18	2.7	7.4	43.0
